# Alternative Therapeutic Interventions: Antimicrobial Peptides and Small Molecules to Treat Microbial Keratitis

**DOI:** 10.3389/fchem.2021.694998

**Published:** 2021-08-11

**Authors:** Praveen Kumar Jadi, Prerana Sharma, Bharathi Bhogapurapu, Sanhita Roy

**Affiliations:** ^1^ Prof, Brien Holden Eye Research Centre, LV Prasad Eye Institute, Hyderabad, India; ^2^ Department of Animal Sciences, University of Hyderabad, Hyderabad, India

**Keywords:** ocular surface, wound healing, microbial keratitis, antimicrobial pepides, small molecules, drug delivery systems

## Abstract

Microbial keratitis is a leading cause of blindness worldwide and results in unilateral vision loss in an estimated 2 million people per year. Bacteria and fungus are two main etiological agents that cause corneal ulcers. Although antibiotics and antifungals are commonly used to treat corneal infections, a clear trend with increasing resistance to these antimicrobials is emerging at rapid pace. Extensive research has been carried out to determine alternative therapeutic interventions, and antimicrobial peptides (AMPs) are increasingly recognized for their clinical potential in treating infections. Small molecules targeted against virulence factors of the pathogens and natural compounds are also explored to meet the challenges and growing demand for therapeutic agents. Here we review the potential of AMPs, small molecules, and natural compounds as alternative therapeutic interventions for the treatment of corneal infections to combat antimicrobial resistance. Additionally, we have also discussed about the different formats of drug delivery systems for optimal administration of drugs to treat microbial keratitis.

## Introduction

Microbial keratitis is a leading cause of blindness in India and globally and is a major public health issue that compromises the quality of life. The early symptoms include redness, extreme pain, light sensitivity, and reduced vision. According to the World Health Organization (WHO), corneal infections are likely to cause 1.5–2 million new cases of blindness per year (Whitcher et al., 2001). The risk factors include ocular trauma and injuries, commonly caused by vegetative matters in the developing world. A study found that almost 70% of microbial keratitis cases in South India resulted from ocular trauma and 63% of all patients were involved in agriculture (Bharathi et al., 2007). Alternatively, the increased use of contact lens and self-diagnosed use of corticosteroids in the modern urban livelihood are the contributing risk factors in the developed world. Studies conducted in France and Sweden showed contact lens wear as a major risk factor in 50% of all keratitis cases (Sagerfors et al., 2020). Bacterial and fungal infections constitute two major forms of corneal infections, consisting of 60 and 39%, respectively (Das et al., 2019). The most common bacterial species responsible for causing corneal infections are *Pseudomonas aeruginosa* and *Streptococcus* spp. along with *Staphylococcus aureus*, *S. epidermidis, Serratia marcescens*, and *Haemophilus influenza* (Bremond-Gignac et al., 2011; Paradiso et al., 2016; Chojnacki et al., 2019; Urwin et al., 2020). In contrast to bacterial keratitis, fungal keratitis occurs widely in the tropical and subtropical climates, and *Aspergillus* spp. and *Fusarium* spp. are the most common etiological agents. Fungal infections of the cornea need to be promptly recognized to facilitate a complete recovery as it is often difficult to treat due to penetration of the hyphae into the stroma. Infection with *Fusarium* spp. often completely destroy an eye in a few weeks, since the infection is usually severe, and perforation may supervene (Thomas, 2003). Studies have shown that almost 42–60% of corneal infections due to *Aspergillus* spp. lead to penetrating keratoplasty (Leck et al., 2002).

**FIGURE 1:**
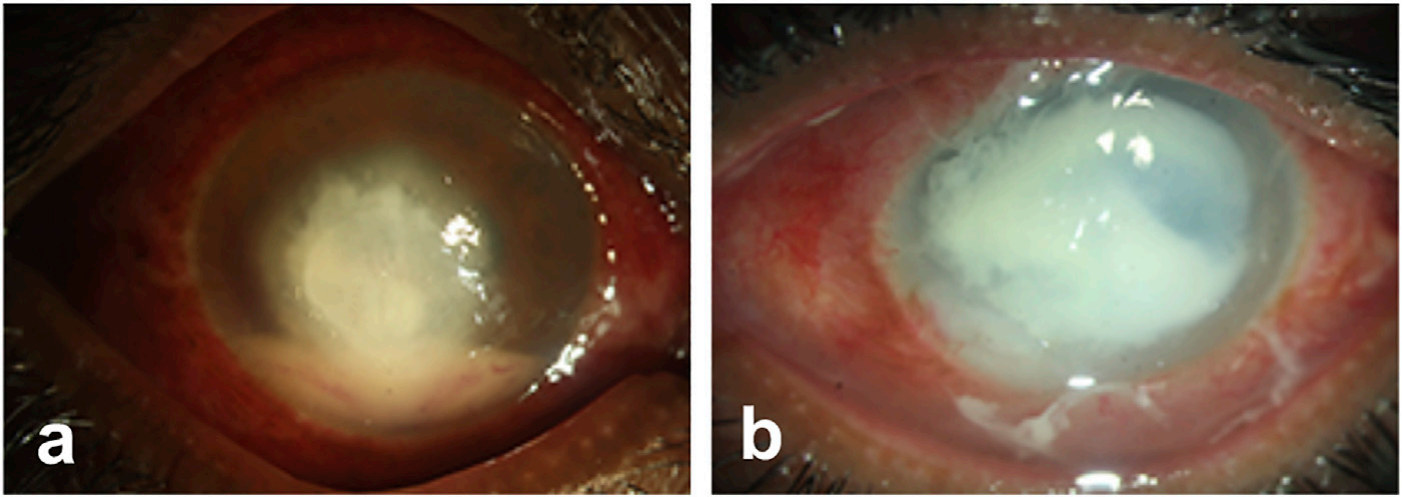
Representative images of microbial keratitis. The pathogens causing infections penetrates deep into the stroma inducing inflammation and corneal opacity that leads to loss of vision.

The representative image of corneal infections caused by *A. flavus* (Figure 1A) and *P. aeruginosa* (Figure 1B) shows increased inflammation and corneal opacity associated with microbial keratitis. Bacterial infections are commonly treated by topical, or subconjunctival administration with penicillin, fluoroquinolones, tetracyclines, and aminoglycosides (Sharma, 2011; Dubald et al., 2018). However, bacteria are gaining resistance against available antibiotics due to the misuse or prophylactic use of antibiotics as well as adaptation of bacteria to the available therapeutics. Similarly, a parallel rise in the number of drug-resistant fungal species is also reported lately. Therefore, researchers are trying to develop new therapeutics or exploring the natural compound to inhibit microbial infections and combat antimicrobial resistance (AMR).

In this review, we have discussed the important role of antimicrobial peptides (AMPs), natural compounds, and small molecules in combating corneal infections. Additionally, we have explored the importance of drug delivery systems in delivering the therapeutics for the treatment of microbial keratitis.

## Methods

We used online literature databases like PubMed and Scopus for searching relevant research articles based on the specific keywords. The keywords used for search included “keratitis”, “antimicrobial resistance”, “antimicrobial peptides”, “small molecules”, “drug delivery systems for delivering antimicrobial drugs,” and “wound healing”. The results were narrowed down by selecting articles relevant to the current review. The Food and Drug Administration (FDA) website was searched for the list of approved antimicrobial peptides in clinical use.

## Antimicrobial Resistance and Limitations of Antimicrobial Drugs

Currently, ocular infections due to bacteria are managed using antibiotics like fluoroquinolones, vancomycin, cefazoline, bacitracin, sulfamethoxazole, erythromycin, chloramphenicol, and aminoglycosides (Lin et al., 2019). However, ever since the discovery of the first antibiotic penicillin, bacteria have shown to develop resistance against them in no time (Fairbrother, 1956). The Antibiotic Resistance Monitoring in Ocular Microorganisms (ARMOR) surveillance program, in its recent antibiotic resistance reports on 4,829 isolates, showed that *S. aureus* strains, including methicillin-resistant *S. aureus* (MRSA), are resistant to moxifloxacin (Thomas et al., 2019). In a recent report it was found that isolates of *S. aureus* and *S. pneumoniae* causing infectious keratitis show reduced susceptibility to fluoroquinolones (Sagerfors et al., 2020). Other reports found that *Corynebacterium* spp. present on the ocular surface were resistant to quinolones (Aoki et al., 2021) and macrolides in addition to fluoroquinolones (Hoshi et al., 2020). According to the World Health Organization (WHO), several bacteria like *Klebsiella pneumoniae*, *E. coli*, *S. aureus*, and *Neisseria gonorrhoeae* show resistance toward antibiotics like ciprofloxacin, carbapenem, fluoroquinolone, methicillin, third generation cephalosporins, sulphonamides, penicillin, tetracyclines, macrolides, fluoroquinolones, and early generation cephalosporins. Earlier, the WHO had published a list of pathogens developing resistance that require immediate attention for the development of new antimicrobials against them and *Acinetobacter baumannii* and *P. aeruginosa* were listed as “critical” priority targets (Shrivastava et al., 2018). It is predicted that *P. aeruginosa* causing ocular infections can develop resistance against beta-lactam, aminoglycoside, and fluoroquinolones. Genetic mutations and inter/intra-species transfer of mobile genetic elements was found to be the major cause of antibiotic resistance spread (Subedi et al., 2018). In another study, 42.9% of *P. aeruginosa* and 57.1% of *Staphylococcus spp*. isolated from corneal ulcers exhibited multidrug (MDR) resistance (Heidari et al., 2018). It was also found that MDR and MRSA isolates are increasing in serious ocular infections (Asbell et al., 2008). Ciprofloxacin and levofloxacin resistance among methicillin-sensitive *S. aureus* was also found frequently in corneal and conjunctival isolates (Marangon et al., 2004). Isolates of *P. aeruginosa* and *S. aureus* were also found to be resistant to ciprofloxacin, gentamicin, and cephalosporins (Willcox, 2011). Currently for management of MDR bacteria, antibiotics like linezolid, colistin, and imipenem are used (Egrilmez and Yildirim-Theveny, 2020). However from what we know about earlier antibiotics, the development of resistance against these antibiotics in bacteria may occur anytime soon. Similarly, the repertoire of effective antifungal agents remains very limited, with only three classes of drugs available for systemic therapy, polyenes (e.g. amphotericin B), triazoles (e.g. fluconazole), and echinocandins (e.g. caspofungin) (Nami et al., 2019). Fluconazole and ketoconazole show better intraocular penetration and acts against keratitis with deep lesions and are considered effective against both *Candida* and filamentous fungi (Chang and Chodosh, 2011). Natamycin is effective when it is topically administered; however, only 2% of the total drug is bioavailable in the cornea (O'day et al., 1986). *Scedosporium* spp. show resistance against amphotericin *in vitro* and both amphotericin B and miconazole show variable activity against *Fusarium* spp. (Klepser, 2011). Ketoconazole and itraconazole have poor *in vitro* activity against *Aspergillus* and *Fusarium* spp. (Pearce et al., 2009)*.* Hence, several alternative therapeutic approaches are explored for the management of ocular microbial infections, some of them include corneal collagen cross-linking (Price and Price, 2016; Naranjo et al., 2019), photodynamic antimicrobial therapy (PDAT), and the use of antimicrobial peptides (AMPs), including host defense peptides and synthetic peptides. Alternative strategies are also required to develop antifungals as the development of drug resistance and limited availability of antifungals represent the main concerns for the current antifungal treatments. The major advantages of AMPs as an alternative are their broad-spectrum activity, rapid killing, anti-biofilm, lower chance of development of resistance, and low cytotoxicity to host. In the following sections, we have provided detailed description of AMPs and their protective role in preventing corneal keratitis.

## Antimicrobial Peptides

Antimicrobial peptides are a diverse group of small proteins that acts as the first line of defense and mounts an attack against invading pathogens. They are found to be evolutionarily conserved in the genome and synthesized by all forms of life (Hancock, 2000)*.* In nature, AMPs are produced either by ribosomal translation of mRNA or by non-ribosomal peptide synthesis (Hancock and Chapple, 1999). In mammals, they are mainly found within granules of neutrophils; however, epithelial cells in the cornea, skin, or other mucosal sites also secrete AMPs. Often, several AMPs with different modes of action are encoded in a cluster of the genome and are co-expressed resulting in the accumulation of several AMPs at a specific site (Lai and Gallo, 2009). Due to their distinguished mode of actions, they are often effective against MDR isolates. Several AMPs are often produced in a catalytically inactive form and needs proteolytic cleavage to become active (Bals, 2000). AMPs are commonly classified based on their secondary structures as *α*-helix, *β*-sheets, and random coil that lack a secondary structure. Antimicrobial peptides approved by the Food and Drug Administration (FDA) for clinical trials are listed in Table 1.

**TABLE 1:** Antimicrobial peptides in clinical trial.

AMP	Source	Target	Phase	References
hLF1-11	Lactoferricin derivative	MRSA, *K. pneumoniae*, *L. monocytogenes,* fungal infections	I/II	Morici et al. (2016)
PAC113	Histatin-5 analogue	Oral candidiasis	II completed	Mohammad et al. (2015)
POL7080	Protegrin analogue	*P. aeruginosa, K. pneumoniae*	III	Butler et al. (2013)
LTX-109 (Lytixar)	Synthetic peptide	Gram-positive MRSA skin infections	IIa completed	Mohammad et al. (2015)
LL-37	Human cathelicidin	Leg ulcer	IIb	Boparai and Sharma (2020)
Novexatin (NP213)	Cyclic cationic peptide	Fungal nail infection	IIb	Javia et al. (2018)
D2A21	Synthetic peptide	Burn wound infections	III	Ballweber et al. (2002)
SGX942	Synthetic peptide	Oral mucositis	III	Butler et al. (2017)
PXL01	Lactoferrin analogue	Postsurgical adhesions	III	Wiig et al. (2011)
OP-145	LL-37 derivative	Chronic middle ear infection	Phase II completed	Ming and Huang (2017)
Mycoprex	Extracted from insects	Fungal infections	III	Hancock (2000)
Murepavadin (POL7080)	Amino acid substitution of protegrin I	Nosocomial pneumonia and ventilator-associated bacterial pneumonia (VABP)	III	Butler et al. (2017)
OP-145	Synthetic 24-mer peptide derived from LL-37	Antibacterial, Chronic bacterial middle ear infection	II completed	Ming and Huang (2017)
Histatin	Naturally occurring peptide	Oral candidiasis	Phase II/III	
MX-226	Cationic Peptide	Dermatology related infections	Phase III b	Felício et al. (2017), Boparai and Sharma (2020)
HB-107	Cecropin B derivative	Wound healing	Preclinical	Mangoni et al. (2016)
PL-5	Alpha helical synthetic peptide	Treatment of skin infections	Phase II	Feng et al. (2015)
Daptomycin	*Streptomyces roseosporus*	Gram-positive bacteria	In market	Cortes-Penfield et al. (2018)

### Antimicrobial Peptides in Eye

**FIGURE 2:**
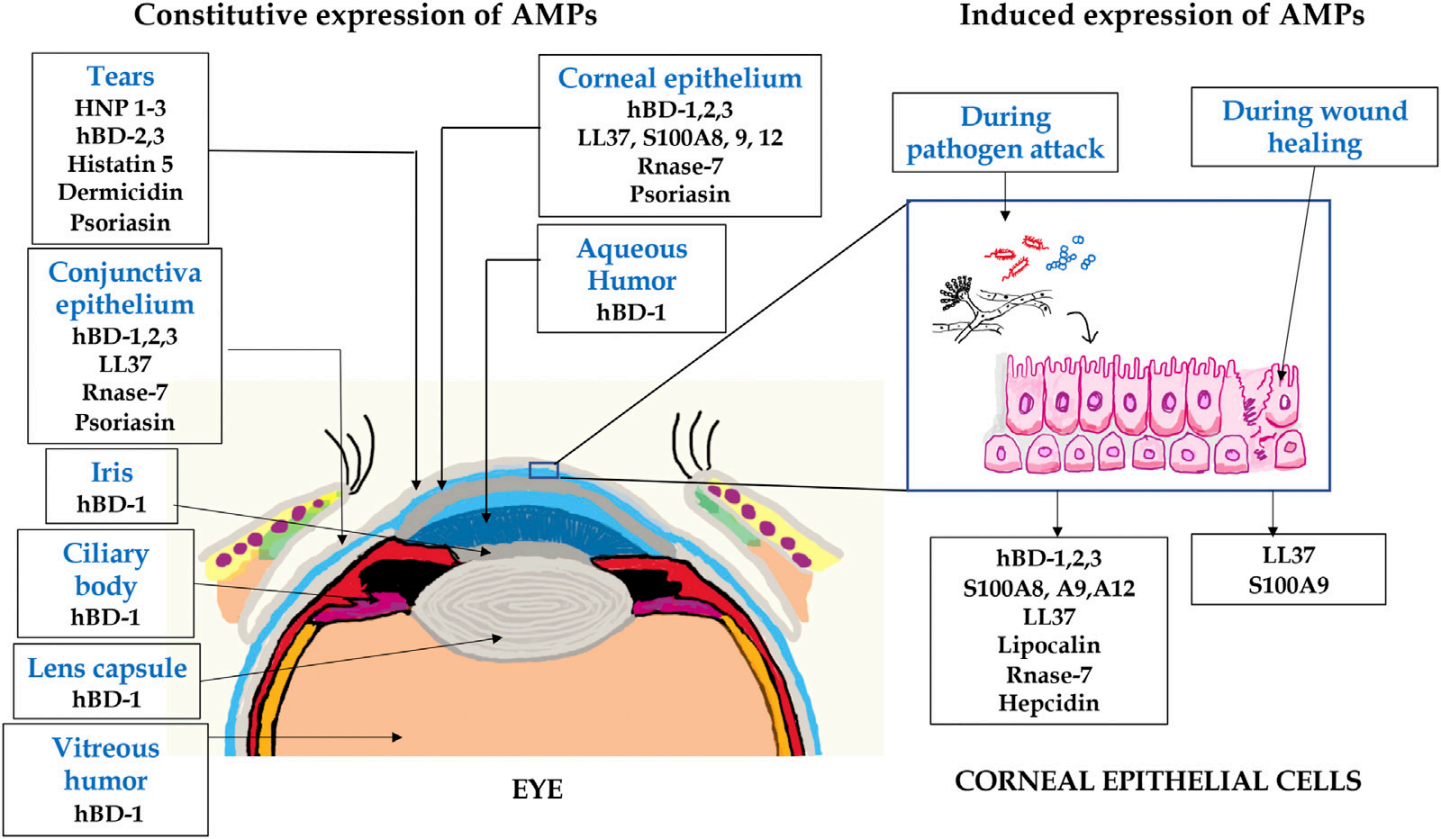
A schematic representation of AMPs expressed in eye. The expression of several AMPs in the anterior part of the eye including tears, conjunctiva, iris, lens, aqueous humor, and corneal epithelium. The corneal epithelial cells express several AMPs constitutively or in response to pathogen attack and wound healing.

**FIGURE 3:**
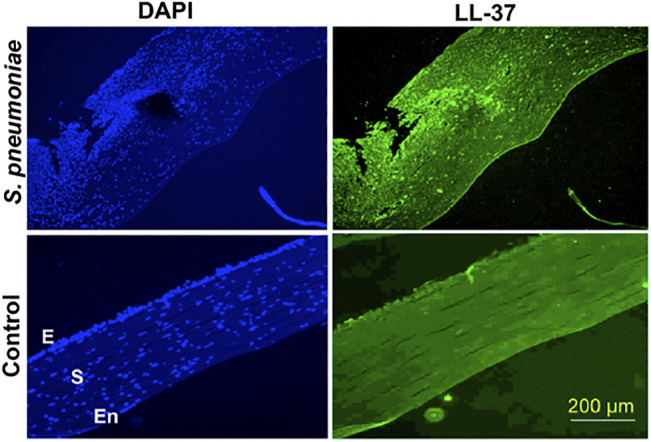
LL-37 expression in corneal tissues obtained from *S. pneumoniae* keratitis patients. Corneal sections of keratitis patients obtained during corneal transplantation and control cadaveric corneas were stained with anti-LL-37 antibody, followed by Alexafluor 488 secondary antibody and imaged under a fluorescent microscope using 10X objective. The sections were counterstained with DAPI. E denotes epithelium, S-stroma, and En-endothelium. This figure is reprinted from reference Sharma et al. (2019).

The cornea and ocular surface are protected from trauma and infection by physical and molecular defenses. The first effective defense involves eyelid closure and blinking, which protects the cornea from physical trauma and removes microbes from the ocular surface. In addition, ocular surface mucins and the tear film restrict pathogen interaction with the corneal epithelium (Mantelli and Argueso, 2008). Corneal epithelium is multilayered in nature and acts as the first line of defense during pathogen attack. Cornea expresses several AMPs (Figure 2) that have microbicidal properties or aids in wound healing, either constitutively or in response to microbial antigens (Mc et al., 1999; Mcdermott, 2009; Sharma et al., 2018; Sharma et al., 2019). β-defensins are produced by the ocular surface and α defensins are supplied into the ocular surface fluids by resident or passing neutrophils and lacrimal ductular epithelia secretion (Kumar et al., 2006). Defensins are 3–4 kDa cationic AMPs that are ubiquitous in nature and its structure contains six cysteine residues paired by disulfide bonds (Ganz and Lehrer, 1994). β-defensin-1 to -4, liver expressed antimicrobial peptide (LEAP)-1 and -2, and LL-37/cathelicidin are often found in ocular surface epithelia (Paulsen et al., 2001). LEAP-1 is a cationic peptide made up of twenty five amino acids and contains four disulfide bonds (Krause et al., 2000). LL-37 is another cationic peptide consisting of 37 amino acids and is the only member of human cathelicidin family (Durr et al., 2006). Human β-defensin (hBD)-1 is constitutively expressed in corneal and conjunctival epithelial cells whereas, in contrast, hBD-2 is rarely expressed in normal tissues. The corneal epithelial cells also constitutively express hBD-3, which some have found to be upregulated in response to cytokines (Mcdermott et al., 2003). hBD-4 is often found to be expressed *in vitro*, but rarely *in vivo*. hBD-9 is expressed by corneal and limbal epithelia and corneal stroma at modest levels, whereas conjunctival epithelium shows the presence of high levels of hBD-9 protein (Mohammed et al., 2010). LL-37 is expressed by both corneal and conjunctival epithelial cells (Gordon et al., 2005) and along with β-defensins contribute to the ability to resist pathogen attack by these cells (Augustin et al., 2011). S-100 class of proteins or calgranulins comprises a group of calcium-binding peptides that usually function as dimers (Fano et al., 1995). Constitutive expression of S100A7 has been reported in cornea, conjunctiva, nasolacrimal ducts, and lacrimal glands. S100A8 and S100A9 were also found to be expressed by human corneal stromal cells (Wilkinson et al., 2016). Ocular fluid also contains human neutrophil peptides (hNP-1 and -2), hBD-1, hBD-2, hBD-3, lactoferrin, lysozyme, and transferrin during normal conditions (Silva et al., 2013). Lactoferrin, a member of transferrin family, is an iron-binding protein having different isoforms such as lactoferrin α, β, and γ (Anderson et al., 1990) and plays an important role in human innate immune system (Baveye et al., 1999). Transferrin is another iron-binding glycoprotein that is responsible for the transport of iron in biological fluids (De Jong et al., 1990). Lysozyme, a 15 kDa protein, present abundantly in tears hydrolyses bacterial cell wall (Mcdermott, 2013). The tear film also contains lactoferrin, β-defensins, calprotectin, and lipocalin (Dartt, 2011; Rusciano et al., 2018). Though their role in tears is not fully known, it is likely that cation (Fe^2+^ and Zn^2+^) sequestration in the ocular surface inhibits fungal germination and growth, which requires these essential metals. These constitutively expressed AMPs have a major role to play in the prevention of infections and aids in host defense. Murine β-defensin 3 (mBD-3, an orthologue of hBD-2) contributes to the clearance of *P. aeruginosa in vivo* and additionally limits *P. aeruginosa* adhesion to the corneal epithelial surface, thus suggesting that AMPs are also involved in maintaining normal resistance to infection in the healthy tissues (Augustin et al., 2011). hBD-2 is found more frequently in infected and inflamed ocular surface tissues. Bacterial products such as lipopolysaccharide (LPS) and pro-inflammatory cytokines such as interleukin (IL)-1β have been shown to upregulate hBD-2 expression in corneal and conjunctival epithelial cells *in vitro* through mitogen-activated protein (MAP) kinase and NF-κB pathways. Differential expression of several AMPs was identified in patients with *P. aeruginosa* and *S. pneumoniae* corneal infections (Sharma et al., 2018; Sharma et al., 2019). Increased expression of LL-37 was also detected in the corneal tissues obtained from patients with *S. pneumoniae* keratitis (Figure 3) (Sharma et al., 2019). hBD-9 was found to be downregulated in bacterial keratitis caused by both Gram-positive and Gram-negative bacteria (Mohammed et al., 2010). hBD-3 and RNAse7 were found to be significantly increased in corneal epithelial cells in response to *A. castellanii* (Otri et al., 2010). A significant increase in the expression of S100A8, S100A9, and hBD-1 was observed both *in vitro* and in corneal ulcers of patients during the *Corynebacterium pseudodiphtheriticum* infection (Roy et al., 2015). Mohammed et al. (2020) observed increased expression of hBD-1, 2, and 9 in patients with fungal keratitis. However, we have found reduced expression of β-defensins in patients with *A. flavus* corneal infections (Mallela et al., 2021).

### Natural Peptides

Peptides are broadly classified into two classes based on their origin, natural peptides, and synthetic peptides. Natural peptides are secreted by a large number of different species such as bacteria, virus, amphibians, mammals, insects, and plants. These peptides can act as natural ligands either as agonists or antagonists and are highly selective and well tolerated. Bacterial infection causes upregulation of several host defense peptides that helps to combat the attack of pathogens. hBD-2 was frequently reported in infected/inflamed ocular surface tissues. Increased expression of defensins was also observed in a murine model of corneal infections, and mBD-3 increased resistance to *Pseudomonas* corneal infections and modulate Toll-like receptor (TLR) signaling in the infected corneas (Wu et al., 2009). Ocular commensal like *C. mastidis* has been shown to induce IL-17 expression that elicits the release of AMPs into tears and protect against *P. aeruginosa* infections (St Leger et al., 2017). The topical application of OH-CATH30, an AMP identified in king cobra is efficacious against drug-resistant *P. aeruginosa* keratitis (Li et al., 2014). LL-37 showed potent antibacterial activity against both laboratory strain and ocular clinical isolates of *S. pneumoniae* (Sharma et al., 2019). It also showed antibacterial activity against *S. aureus* and *S. epidermidis*. Cathelicidin deficit mice also showed increased susceptibility to *P. aeruginosa* keratitis (Huang et al., 2007). S100A8/A9 was found to induce reactive oxygen species (ROS) and aided in macrophage-mediated bacterial killing during *P. aeruginosa* keratitis (Deng et al., 2013). Lipocalin present in tear was reported to inhibit both bacterial and fungal growth (Fluckinger et al., 2004) and also suppressed ocular inflammation in LPS-induced uveitis (Tang et al., 2018). The antimicrobial peptide Css54, isolated from the venom of *Centruroides suffuses*, was found to exhibit antimicrobial activity against bacteria such as *Listeria monocytogenes*, *Streptococcus suis*, *Campylobacter jejuni,* and *Salmonella typhimurium* that cause zoonotic diseases (Park et al., 2020). Using a high-throughput technique of the phage display method, 12-mer peptide phage display library was screened to identify peptides effective against infectious keratitis. Both Pc-C and Pc-E peptides inhibit the adhesion of *A. fumigatus* to human corneal epithelial cells (HCEC) and decreased fungus-mediated corneal disruptions in a mice model of keratitis (Zhao et al., 2012). Endogenous expression of mBD3, mBD4, and CRAMP was found to play an important role in *F. solani* keratitis (Kolar et al., 2013). Increased expression of LL-37, hBD-2, and hBD-3 in corneal epithelial cells was also noted in response to heat-killed *C. albicans* (Hua et al., 2014). Cullor et al. (1990) found defensins, NP-1, and NP-5, effective against *C. albicans’* ocular isolates found in humans and horses. A study by Sengupta et al. (2012) reported the antifungal activity of lactoferricin B against fungal pathogens and its effect on eradication of the biofilms from contact lenses. Lactoferricin B helps to decrease the dosage of antifungal agents by 8 folds against biofilms formed by *A. fumigatus, F. solani*, or *C. albicans*. Another study reported that α defensins are known to show strong antifungal activity against *A. fumigatus*, *C. neoformans,* and *C. albicans* (Delliere et al., 2020). Histatin -5 is a histidine-rich peptide which is produced by salivary glands in humans and higher primates (Du et al., 2017). Both hNP-1 and histatin-5 were found in human saliva exhibiting candidacidal activity due to their non-lytic release of mitochondrial ATP and chelation of the metal ions (Helmerhorst et al., 2001). hBD-1, -2, -3, histatin-5, and LL-37 were found to show fungicidal activity against *Candida* spp. *via* secreted glycosylated exodomain protease, Msb2 (Swidergall et al., 2013). Human cathelicidin and β-defensins permeabilize the cell membrane of the yeast that leads to the death of the fungi (van Der Weerden et al., 2010). HBD-3 and LL-37 bind to the β-1, 3-exogluconase of the cell wall of *C. albicans*, thereby reducing its infectivity toward the host (Mohammed I. et al., 2017). Histatin-5 kills the fungus by different mechanisms, it activates the stress response pathways and secretion of the proteases. Histatin-5 enter into the fungus *via* Dur3/Dur 31 transporters, induce ATP efflux and release of ROS, and finally activate the high osmolarity glycerol stress response pathway, followed by extrusion of histatin-5 *via* efflux transporter, Flu1 (Edgerton et al., 2000). RNase-3 and RNase-7 were known to show fungicidal activity at lower micromolar concentration against *C. albicans* by perturbing the cellular RNA and disturbing the stability of the cell membrane (Berman, 2012). A reduced form of psoriasin (S100A7), a linear peptide, showed killing activity against *A. fumigatus* by chelation of zinc metals and leading to fungal apoptosis (Mohammed I. et al., 2017). Calprotectin (S100A8/A9) exhibited antifungal activity, blocked hyphal growth, and limited the growth of *Aspergillus* spp. by inhibiting transport of fungal zinc and manganese in a mouse model of keratitis (Clark et al., 2016).

### Synthetic Peptides

Synthetic or semi synthetic peptides are generally designed to upregulate the pharmacological properties and to overcome the limitations of naturally derived AMPs including host toxicity, degradation by proteases, and loss of antimicrobial activity in presence of physiological salt concentration (Mohamed et al., 2016). The short synthetic peptides have high electivity and reduced cost of production making them ideal for translational clinical application. Several peptides designed and synthesized have been tested against various bacteria and fungus both *in vitro* and *in vivo*. EC1-17KV, synthetic peptides based on the antimicrobial peptide EeCentrocin 1 from *Echinus esculentus*, was efficient in killing MDR *P. aeruginosa* and exhibited multiple modes of action, including direct membrane disruption and inhibitory effects on cell adhesion and biofilm formation (Ma et al., 2020). Another novel short α-helical hybrid peptide inspired by natural α-helical AMPs, PA-13, showed remarkable broad-spectrum antibacterial activity, especially against *P. aeruginosa* with no toxicity to mammalian cells. It displayed rapid binding and penetration activity which resulted in membrane permeabilization and also exhibited an anti-inflammatory response by neutralizing LPS (Klubthawee et al., 2020). Pam-3, a palmitoleic acid–modified octapeptide fragment designed from hBD-1, was found to be effective *in vitro* against multidrug-resistant ESKAPE pathogens (six nosocomial pathogens, *Enterococcus faecium*, *S. aureus*, *K. pneumoniae*, *A. baumannii*, *P. aeruginosa*, and *Enterobacter species* that exhibit multidrug resistance and virulence). Pam-3 is also highly effective against *A. baumannii* resistant to the last-resort antibiotics, colistin and tigecycline. Additionally, Pam-3 was able to eradicate established biofilms formed by *S. aureus* and *P. aeruginosa in vitro* (Koeninger et al., 2021). SAAP-148, a synthetic LL-37 inspired peptide, is highly effective against MDR Gram-positive and Gram-negative ESKAPE pathogens, as well as isolates of *E. cloacae*, *E. coli*, and *K. pneumoniae* resistant to the last-resort antibiotic colistin. SAAP-148 was also able to kill *Clostridium difficile* under anaerobic conditions and successful in preventing the formation and eradication of established biofilms by *S. aureus* and *A. baumannii* (De Breij et al., 2018). A synthetic peptide, TC19, derived from the human thrombocidin-1–derived peptide L3, showed efficient and rapid killing in human plasma of MDR strains of several ESKAPE bacterial species. FK16, a cathelicidin-derived shorter peptide, was found to show increased killing ability toward *P. aeruginosa*, by itself or in synergy with vancomycin without having any toxicity to the host (Mohammed et al., 2019). A hybrid peptide of cecropin A and mellitin (derived from venom of European honey bee) was found to be effective against *P. aeruginosa* in a rabbit model of keratitis (Nos-Barbera et al., 1997). Cationic antimicrobial protein (CAP37)–derived peptide analogues showed significant antifungal activity against multifarious *Candida* species from patients with vulvovaginitis. Bactericidal permeability-increasing (BPI) protein, a cationic protein derivative has broad spectrum activity against many fungi, including *Candida spp*., *C. neoformans*, and *A. fumigatus*. Shorter synthetic peptides (XMP.284, XMP.366, and XMP.39 1) which are derivatives of BPI protein domain III analogs act against *Candida spp*. When these peptides were used in combination with flucanozole, the minimum inhibitory concentration (MIC) of the antibiotic reduced up to 8-fold. The peptide XMP 391 was effective against murine-disseminated aspergillosis and enhanced the effectiveness of amphotericin B. The peptides ToAP2 (derived from venom of scorpion *Tityus obscurus*) and NDNP-5.7 also showed antifungal properties against *C. albican*s infection in which ToAP2 exhibited a broad spectrum of activity. These peptides show fungicidal nature by permeabilizing the cell membrane and invoked changes in cell morphology such as cell disruption 24-h posttreatment (Do Nascimento Dias et al., 2020). P10, an α-helical amphipathic AMP, exhibited an enhanced antimicrobial spectrum against. *C. albicans* and *A. niger* (Kang et al., 2017). Lipopeptides, which were secreted by the *P. syringae*, was particularly active against several filamentous fungi and yeasts, including *Candida, Cryptococcus,* and *Aspergillus* strains (De Luca and Walsh, 2000). Cecropins, an AMP derived from insects, exhibited antifungal properties against *Aspergillus* and *Fusarium spp*. (Andra et al., 2001). This peptide targets the cell membrane by disrupting it and creates ionic imbalance and intracellular redox states in *C. albicans*. The upregulation of expression of CRAMP, a mouse analogue of human LL-37, leads to a decrease in the gastrointestinal colonization of *C. albicans* (Niyonsaba and Ogawa, 2005). The peptide proteagrin-1 from porcine was particularly active against a broad range of fungi, including several *Candida spp*. (including drug-resistant strains) and *Cryptococcus neoformans* (Buda De Cesare et al., 2020). The same study also reported other peptides like SMAP-29 (cathelicidin in ovine), BMAP-27, and BMAP-28 (two bovine α-helical) to be effective as proteagrin-1 against *C. tropicalis, C. glabrata*, and *C. parapsilosis* (Buda De Cesare et al., 2020).

### Mode of Action of Antimicrobial Peptides

**FIGURE 4:**
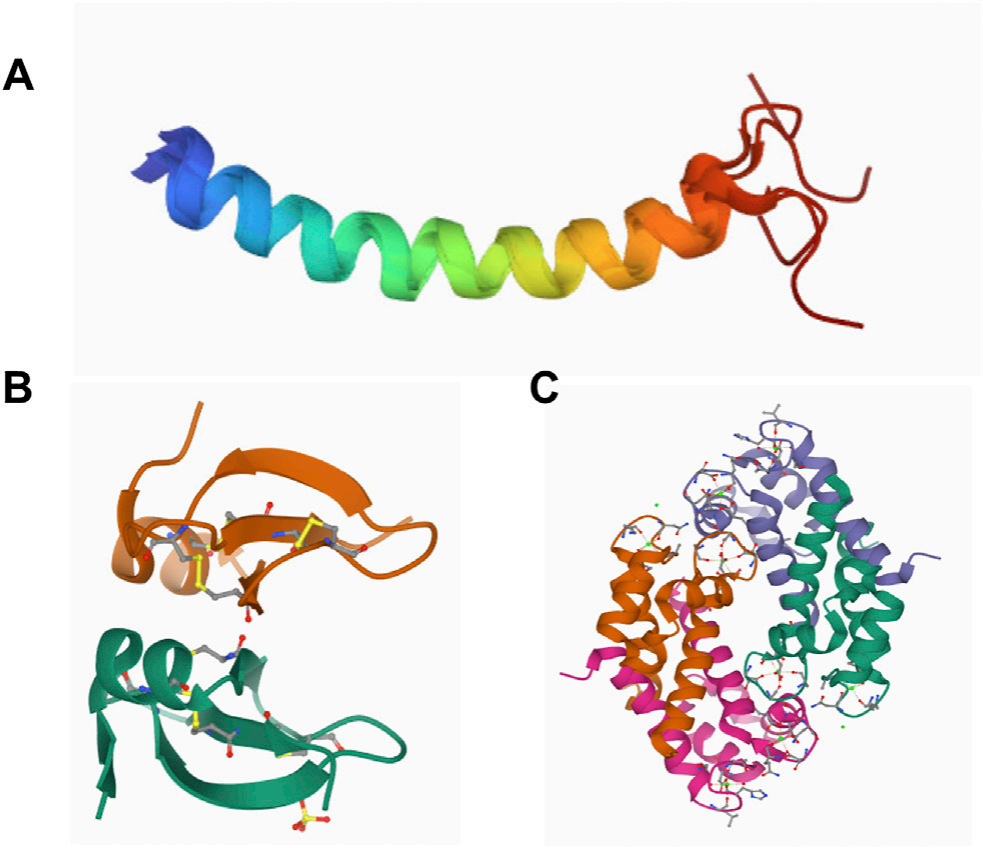
Representative structures of AMPs. Structures of LL-37 (Wang, 2008) **(A)**, hBD-2 (Hoover et al., 2000) **(B)**, and crystal structure of human calprotectin (S100A8/S100A9) (Korndorfer et al., 2007) **(C)**.

In general, AMPs are cationic in nature with a net charge of +2 to +11 and an amphipathic structure that aids in insertion in bacterial cell membrane either as alpha-helices or beta-sheets (Luong et al., 2020). Representative structural images of few AMPs are given in Figure 4. Cationic AMPs selectively bind to the negatively charged surface of the bacterial membrane (Matsumoto et al., 2006; Strahl and Errington, 2017) that differ from mammalian cell membranes consisting of neutral phospholipids (Van Meer et al., 2008). In case of fungi, AMPs inhibit or kill the pathogenic fungi by inhibiting the spore germination or mycelial growth or by causing the structural deformity in the spores/hyphae by creating pores, twists, and making them swollen and broken (Li et al., 2021). Most of the AMPs show a direct action against pathogens by creating pores on their membrane, hence causing leakage and cell death. The models for the creation of pores on the cell membrane by AMPs that have been hypothesized are as follows:

a. Barrel-stave model, where AMPs form bundles and insert in the membrane like a cylinder creating pores, hydrophobic region of peptide interacting with lipid part, and ultimately causing leakage (Bocchinfuso et al., 2009; Zeth and Sancho-Vaello, 2017). For example, dermicidin and GA IV glycotriazole peptides (Junior et al., 2017).
b. Toroidal model, where AMPs arrange and implant themselves vertically in the membrane to make pores and forces the lipids to form micelles and hence disrupts the membrane (Matsuzaki et al., 1995). For example, magainin 2.
c. Carpet-like model, where AMPs form aggregates and arrange parallelly on the membrane like a carpet and intercalate into the membrane, with hydrophobic sites facing the membrane and disrupts the arrangement of the lipid bilayer (Oren and Shai, 1998). For example, LL37 and PMAP-23.

Apart from these classical models, AMPs like R9F2 and (KFF)3K have recently been shown to cause cell wall thinning, loss of cytoplasm structure, formation of mesosome-derived multimembrane structures, and "decorated fibers" derived from DNA chains in *S. aureus* (Grigor'eva et al., 2020) instead of pore formation. EP-2, an antifungal peptide isolated from *B subtilis,* can swell and distort the mycelium of the fungi thereby inhibiting the growth of fungi (Wang et al., 2016)*.* As fungal cells are mainly composed of polysaccharides, β-glucans, chitin, and mannan, AMPs inhibit the synthesis of these saccharides (Li et al., 2021). Anidulafungin, a synthetic lipopeptide, inhibits the synthesis of 1,3 β-D glucan synthesis in the cell walls of *Candida* and *Aspergillus* (Martin Mazuelos and Rodriguez-Tudela, 2008). Apart from cell membrane disruption, some AMPs like Bac 7, PR-39, and HNP-1 (Cardoso et al., 2019) are also involved in the inhibition of protein biosynthesis or nucleic acid biosynthesis (Hale and Hancock, 2007); some like histatin-5 causes protease activity, others like pyrrhocoricin and drosocin (Nielsen et al., 2021) causes disruption of DnaK activity. Indolicidin, aurein, magainin II, cecropin A, and LL-37 were also found to enhance the essential lipid transportation causing rapid scrambling of the lipid composition leading to an increase in ion transport and activation of lethal signaling processes (Paredes-Gamero et al., 2012). Apart from their direct microbicidal activity, AMPs also help in the chemotaxis of host neutrophils, mast cells, and monocytes, phagocytosis, angiogenesis, and wound healing. AMPs can also trigger mast cell degranulation and lead to increased blood vessel permeability. Hence, AMPs can target a pathogen directly or indirectly, ultimately leading to clearance and healing. It has also been shown that some AMPs at lower concentrations activate apoptosis and hence lead to regulated cell death (Lee and Lee, 2014).

## Other Compounds

During corneal infections, pro-inflammatory cytokines like IL-1β, IL-6, and tumor necrosis factor (TNF)-α are released that triggers different signaling pathways leading to collagen degradation in the stroma as well as necrotic death of keratocytes. These inflammatory damages result in the thinning of corneal stroma followed by scar formation (Yan et al., 2017; Crupi et al., 2019). Studies have reported that the topical antioxidants or corticosteroids or lipid treatment reduced the inflammatory reaction that occurs during acute corneal inflammation and protects the ocular damage (Alio et al., 1995; Lim et al., 2015; Ni et al., 2016a). Here, we will be discussing about the antioxidants, small molecules, lipids, and corticosteroids that were used to protect the eye from fungal, bacterial or bacterial toxin–induced inflammatory damage.

### Antioxidants

The generation of ROS by corneal epithelial cells (CEC) often protects corneas from microbial infections by killing the microbes; however, excess production of ROS induces corneal inflammation and results in irreversible corneal opacification and loss of vision (Ruban et al., 2018). Antioxidants reduce the free radical by donating their electrons and decrease the inflammatory damage of CEC. Various studies have reported the usage of antioxidants to protect the cornea from the microbial-induced inflammatory damage. Resveratrol, a naturally occurring phytoalexin produced in the fruits and leaves of edible plants is known to possess antioxidant and anti-inflammatory activity (Iyori et al., 2008; Speciale et al., 2011). Resveratrol downregulates pro-inflammatory cytokines in cells by suppressing the activation of NF-κB. Marino et al. (2013) found that resveratrol protected the cornea from *S. aureus*–induced inflammatory damage by downregulating the expression of TLR2 and IL-8 in an *ex vivo* model of infection. Tempol (4-hydroxy-2,2,6,6-tetramethylpiperidine-1-oxyl), a membrane permeable molecule known to exhibit antioxidant activity, reduces the reactive oxygen and nitrogen species and enhances the catalytic activity of catalase (Batinić-Haberle et al., 2010; Wilcox, 2010). Crupi et al. (2019) used this potent antioxidant molecule to explore its protective effect against LPS-induced corneal cell damage and found that tempol protected the corneal cells from inflammatory damage by enhancing the antioxidant activity of SOD and lowering the prostaglandin E2 (PGE2) levels, cytokines, and COX-2 expression. Asiatic acid (a triterpenoid) was found to protect HCEC from the LPS-induced inflammatory damage by inhibiting p-Akt activation, downregulating inflammatory cytokines, and upregulating antioxidant activity (Chen et al., 2017). Quercetin is a natural flavonoid known for antioxidant activity along with anti-inflammatory, antitumor, and cardiovascular protective activity (Li et al., 2016). Previous studies have reported that quercetin exerts antifungal activity against wide variety of fungal strains (Rocha et al., 2019) and also reported the suppression of LPS-induced retinal inflammation in mice (Ho et al., 2020). Yin et al. (2021) reported protective effect of potential quercetin against *A. fumigatus*–induced corneal keratitis. *In vitro* studies showed that quercetin treatment significantly reduced the growth of *A. fumigatus* without affecting the viability of HCEC, whereas in *in vivo* studies, it reduced the fungal load in the cornea with minimal recruitment and infiltration of neutrophils to the corneal stroma (Yin et al., 2021). Baicalein, a flavonoid isolated from *Scutellarin baicalins,* is also known to have an antioxidant activity along with antifungal and anti-inflammatory applications (Zhu et al., 2021). Zhu et al. (2021) have reported the use of baicalein to protect the cornea from *A. fumigatus* infection in a murine model of keratitis. Treatment of infected mice cornea with baicalein significantly reduced fungal growth, biofilm formation, adhesion, and suppressing inflammatory responses *via* downregulating the thymic stromal lymphopoietin (TSLP)/TSLP receptor (TSLPR) pathway (Zhu et al., 2021). Epigallocatechin gallate (EGCG) is a polyphenol obtained from the green tea is known to have good antioxidant and anti-inflammatory activity (Ruban et al., 2018). Topical administration of voriconazole along with EGCG showed the inhibition of *F. solani*–induced keratitis by reducing the fungal growth and inflammatory responses. Systemic supplementation of vitamin C (ascorbic acid) showed reduced corneal opacity caused by infectious keratitis (Cho et al., 2014). Vincamine, an alkaloid with strong antioxidant activity, protected HCEC against LPS-induced oxidative and inflammatory damage by scavenging ROS and reducing the expression of IL-1β, IL-6, IL-8, TNF-α, and the transforming growth factor (TGF)-β (Wu et al., 2018). Antioxidants having anti-inflammatory activity have advantages in protecting the cornea from the microbial toxin–induced inflammatory damage during corneal keratitis.

### Small Molecules

**FIGURE 5:**
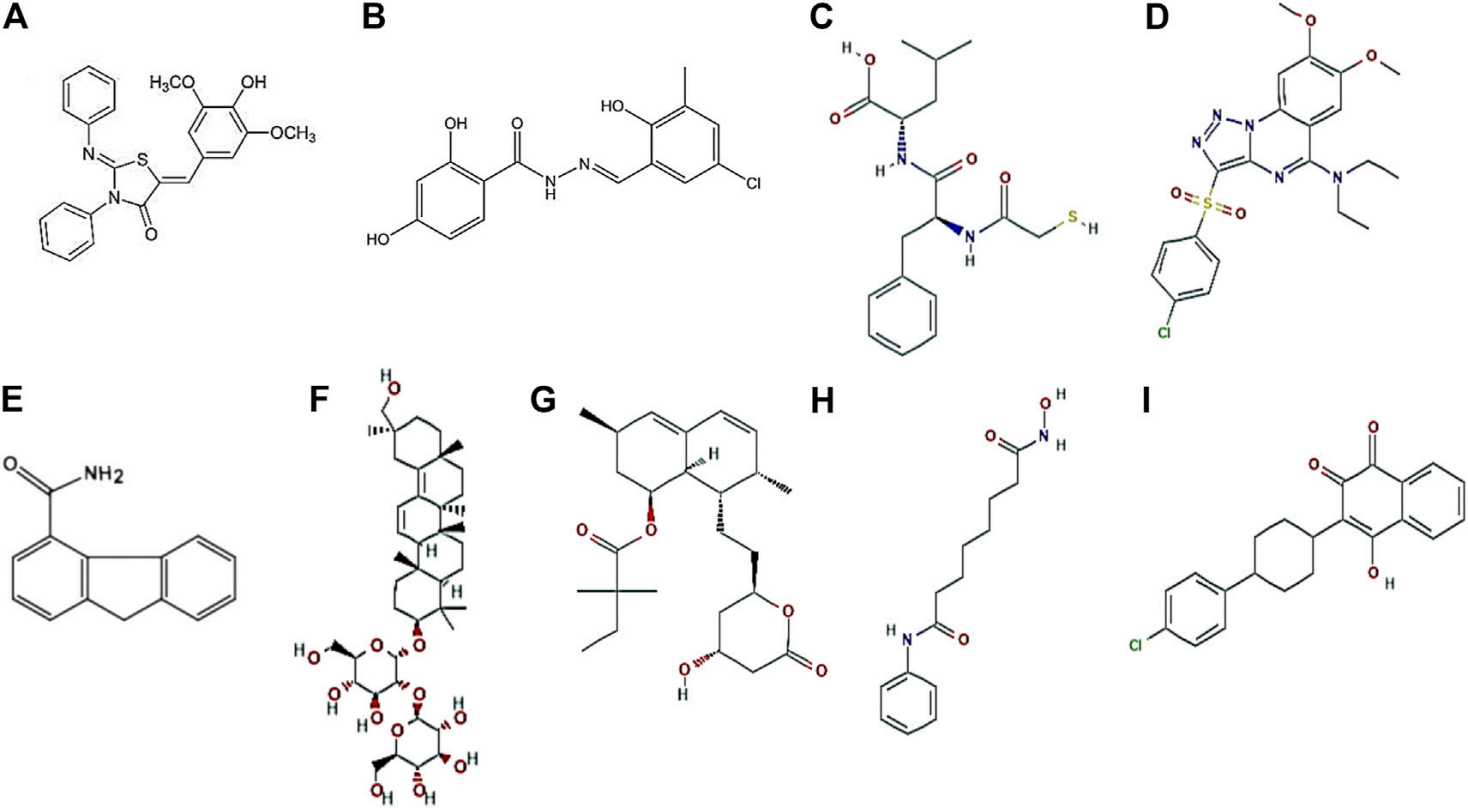
Representative structures of small molecules. 2-Imino-5-Arylidene thiazolidinone (Felise et al., 2008) **(A)**, INP0341 (Nordfelth et al., 2005) **(B)**, 2-mercaptoacetyl-L-phenylalanyl-l-leucine (Kessler et al., 1982) **(C)**, Targocil (Farha et al., 2015) **(D)**, Pseudolipasin A (Lee et al., 2007) **(E)**, Glycyrrhizin (Ji et al., 2016) **(F)**, Simvastatin (Jensen et al., 2016) **(G)**, Suberoylanilide hydroxamic acid (Choi and Pflum, 2012) **(H)**, and Atovaquone (Nixon et al., 2013) **(I)**.

To fight the antibiotic resistance that is increasing rapidly on a global scale, the virulence factors of the pathogens are often targeted and explored as alternative therapeutic agents. High-throughput screening are being widely used to identify various compounds that could be developed as small molecule tools to study the pathogen further or as a treatment for preventing infections. Dajcs et al. have reported the inhibitory role of lysostaphin, a zinc metalloproteinase obtained from *S. simulans*, against *S. aureus*–induced corneal keratitis. *In vivo* studies (rabbit models) displayed that lysostaphin treatment significantly killed more methicillin resistant *S. aureus* (MRSA) or methicillin sensitive *S. aureus* on the corneal surface and the corneal tissues than in vancomycin-treated or untreated eyes (Dajcs et al., 2000). Further, the authors also evaluated possible allergic reactions toward lysostaphin and found no adverse reactions (Dajcs et al., 2002). Small molecule like 2-Imino-5-arylidene thiazolidinone was found to inhibit type II secretory system of *P. aeruginosa* and type 3 secretory system (T3SS) in *Yersinia spp*. (Felise et al., 2008). Recently Sharma et al. (2020) have shown that INP0341, a salicyledene acylhydrazide that blocks T3SS, effectively inhibits *Pseudomonas* infection in a murine model of keratitis. The salicyledene acylhydrazides block the T3SS in several pathogens, including *Y. pseudotuberculosis* (Nordfelth et al., 2005; Bailey et al., 2007), *C. trachomatis* (Slepenkin et al., 2011)*, S. enterica*, and *P. aeruginosa.* Several small-molecule compounds that inhibit *Pseudomonas* elastase have been reported by various groups (Burns et al., 1990; Cathcart et al., 2011; Konstantinovic et al., 2020). The compound 2-mercaptoacetyl-L-phenylalanyl-l-leucine, effectively inhibited elastase activity in rabbit corneas and reduced corneal melting in a rabbit model of *P. aeruginosa* keratitis (Spierer and Kessler, 1984). Targocil, a teichoic acid biosynthesis inhibitor, significantly reduced intracellular growth of *S. aureus* in HCEC and further aided in reducing the adherence of *S. aureus* to corneal cells (Suzuki et al., 2011). Very recently, pseudolipasin A, an inhibitor of ExoU of *P. aeruginosa*, induced scratch healing and reduced cell death of human corneal epithelial cells during *P. aeruginosa* infection (Foulkes et al., 2021). Glycyrrhizin, a glycoconjugate triterpene isolated from *G. glabra*, significantly reduced the adherence of *P. aeruginosa* to corneal epithelial cells and inhibited the growth of bacteria in a mouse model of *Pseudomonas* corneal infections, either alone or in combination with antibiotic (Hazlett et al., 2019). Topical application of simvastatin that blocks HMG-CoA reductase required for siderophore biosynthesis, significantly reduced fungal burden during *A. fumigatus* corneal infections by restricting fungal iron acquisition *in vivo* (Leal et al., 2013). Very recently suberoylanilide hydroxamic acid, a histone deacetylase inhibitor, significantly reduced inflammation and expression of pro-inflammatory cytokines in a murine model of *F. solani* keratitis (Li et al., 2019). Atovaquone, a hydroxynapthaquinone, acts as an antifungal agent by disrupting mitochondrial functions and intracellular zinc storage in *Aspergillus* spp. and *Fusarium* spp. Reduced hyphal growth was observed in murine eyes infected with Fusarium and treated with atovaquone compared to infected eyes (Clark et al., 2018). The represented structures of the small molecules are presented in Figure 5.

### Lipids

The omega-3 fatty acids like docosahexaenoic acid (DHA) and eicosapentaenoic acid (EPA) exhibits anti-inflammatory activity by inhibiting the oxidation of arachidonic acid (AA), which prevents the production of pro-inflammatory molecules like leukotriene B_4_ and prostaglandin E_2_ (Lim et al., 2015). Resolvins, an endogenous lipid mediator derived from EPA (resolvin E, RvE) and DHA (resolvin D, RvD) exhibits anti-inflammatory activity by reducing the production of pro-inflammatory cytokines. Among E-series resolvins, RvE1 possess various biological activities, including antioxidant and anti-inflammatory properties, and also they clear neutrophils from the site of inflammation by recruiting non-inflammatory monocytes and macrophages. This potential molecules was used by Lee et al. (2015) to evaluate its protective effect from bacterial toxin(LPS, antibiotic-killed *S. aureus* and *P. aeruginosa*)–induced corneal inflammatory damage. RvE1 treatment significantly reduced the production of chemokines and pro-inflammatory cytokines in HCEC, human neutrophils (IL-6, IL-8, and CXCL1), murine cornea (CXCL1), and macrophages (CXCL1, TNF-α, and IL-1β). Additionally, RvE1 treatment also reduced the neutrophil infiltrations into the corneal stroma and minimized corneal thickness and haze. RvD1 prevented the corneal inflammatory damage by reducing corneal infiltrates, edema, and production of inflammatory cytokines (IL-6 and CXCL1) along with inhibition of neutrophil recruitment (Lee et al., 2016).

### Corticosteroids

Corticosteroids having anti-inflammatory activity are known to suppress inflammatory pathways in a wide variety of cells by downregulating the inflammatory genes (Tallab and Stone, 2016). Corticosteroids were used for its anti-inflammatory property to treat bacterial keratitis in combination with antibiotics, in addition they also reduce scarring, stromal melts, and neovascularization (Austin et al., 2017; Ni et al., 2016b). However, the use of topical corticosteroids has always been a topic of debate. There are several studies that report use of corticosteroids to be helpful in reducing severity of corneal stromal melt and neovascularization and improve patient compliance by alleviating pain and discomfort. It has also been shown to reduce neutrophil chemotaxis and thus lower cytokine burden. On the contrary, use of corticosteroid in bacterial keratitis has been argued to delay epithelial healing and exacerbation of infection (Gritz et al., 1992). Several animal models were developed to explore the protective effect of corticosteroids from microbial keratitis (Bohigian and Foster, 1977; Badenoch et al., 1985; Gritz et al., 1992). Usage of corticosteroids alone for treating microbial keratitis enhanced the microbial count and extended infection period that leads to the corneal stroma thinning and perforation (Badenoch et al., 1985; Hazlett et al., 2016), whereas, in combination with antibiotics they neither have an effect on antimicrobial activity nor on the final outcome of corneal anatomy (Hindman et al., 2009). Animal studies have also displayed that topical administration of corticosteroids after corneal injury decreased corneal wound healing strength (Sugar and Chandler, 1974). Previous clinical trials have reported the use of corticosteroids in treating corneal keratitis and steroids for corneal ulcers trial (SCUT). A clinical study performed by Carmichael et al. (1990) reported that patients treated with antibiotics only (fortified cefazolin 32 g/L and gentamicin 14 g/L) and antibiotics plus steroids (fortified cefazolin 32 g/L, gentamicin 14 g/L, and 0.1% dexamethasone) showed no statistically significant difference in the outcome in terms of best corrected vision and the ulcer healing rate. A randomized clinical study was carried out by Blair et al. (2011) to investigate the protective effect of antibiotics (gatifloxacin) and antibiotics plus corticosteroids (gatifloxacin plus 0.1% dexamethasone) on the bacterial corneal ulcers. The mean residual ulcers measured by clinician at 10 weeks showed a smaller change in the ulcers size of antibiotics plus corticosteroids–treated patient than the only the antibiotic-treated patients group, though the difference was not significant. A preliminary clinical trial was carried out by Srinivasan et al. (2009) to evaluate the effect of adjunctive topical corticosteroids on the outcome in bacterial keratitis. Patients treated with corticosteroid (prednisolone sodium phosphate 1%) after moxifloxacin (0.5%) showed significant delay in re-epithelialization than control patients (corticosteroids-untreated patients).

## Antimicrobial Peptides and Alternative Therapeutics in Ocular Wound Healing

Corneal wound healing consists of complex sequences of events involving the regeneration of epithelial layers, migration of epithelial cells, fibroblasts for wound closure, and regeneration of tissues in a regulated manner so that the transparency of the cornea is not compromised. Corneal epithelial cells aid in the process by replenishing themselves and mediating cell signaling and crosstalk with stromal cells (Di Girolamo, 2015). The wound healing is facilitated by several growth factors, cytokines, and endogenous AMPs expressed by CEC. Several AMPs that are upregulated in patients with bacterial or fungal corneal infections, like β-defensins, LL-37, S100A8, S100A9, and Reg3γ (Sharma et al., 2018; Sharma et al., 2019), have been shown to also possess wound-healing properties. hBD-2 mRNA was found to be upregulated in the epithelium of wounded corneas and aided in regeneration of corneal epithelium (Mcdermott et al., 2001). hBD-2 also induced expression of several cytokines and enhanced rapid intracellular Ca^2+^ influx that helped in proliferation of keratinocytes in a skin wound model (Mi et al., 2018). Histatin-5 was found to promote cell migration and accelerate wound closure in murine model of corneal epithelial injury (Shah et al., 2020). Increased expression of LL-37 was noted in regenerating corneal epithelium and induced migration of corneal epithelial cells (Huang et al., 2006). Other reports have also shown that LL-37 induced wound healing in the porcine corneal wound model by activation of EGFR signaling *via* PI3K/Akt and ERK signaling pathways (Yin and Yu, 2010). Cationic antimicrobial protein, CAP37, interacts with TLR4 and promotes corneal re-epithelization in mice (Kasus-Jacobi et al., 2020). Very recently, a dipeptide, isolated from human placental extract has been found to accelerate corneal wound healing both *in vitro* and *in vivo* (Nagata et al., 2015). RNase 5 has been shown to facilitate corneal wound healing in rabbit by activation of the PI3K/Akt pathway (Kim et al., 2016). Apart from AMPs, small molecules like Y-27632, a Rho-associated protein kinase inhibitor, has also been shown to induce proliferation of limbal stem cells and improve wound healing in *in vivo* mouse model (Sun et al., 2015) by modulation of cell-cell adhesion. Several lectins have also been shown to promote regeneration of corneal epithelium. KM+, a lectin obtained from *Artocarpus integrifolia*, promotes neutrophil migration and restoration of corneal epithelium in a rabbit corneal wound model (Chahud et al., 2009). Galectin 3 and galectin 7 have also shown to accelerate wound healing in a corneal alkali burn model (Cao et al., 2003). Acacia honey has been reported to accelerate corneal wound healing in rabbit corneal fibroblasts *in vitro* (Abd Ghafar et al., 2016). Another traditional herbal medicine, *Centella asiatica*, helped in proliferation and migration of rabbit corneal epithelial cells (Ruszymah et al., 2012). These data show promises that AMPs or their derivatives and mimetics can be potentially be useful to promote wound healing following corneal damages due to accidental injuries, or various surgeries.

## Ocular Drug Delivery Systems to Treat Keratitis

Treatment of cornea appears to be easily accessible; however, various physicochemical barriers hinder the drug penetration into this tissue. In addition to this, other factors, like excess tear secretions, blinking reflex, and nasolacrimal drainage system, significantly reduces drug retention time in the ocular surface; only 1–7% of the available drug reaches the corneal stroma (Hewitt et al., 2020). Therefore, a high dosage of the drugs are required for treating the infections, and nasolacrimal regions exposed to those drugs lead to adverse side effects (Phan et al., 2014; Djebli et al., 2017). In order to minimize high usage of drugs and reduce their side effects, the drugs need to be delivered to the site of infection. Drug delivery systems play a critical role in delivering the drugs to the infected sites. In this section, we will discuss the different types of drug delivery systems for delivering therapeutics to treat corneal infections.

### Contact Lens

Contact lenses (CLs) are transparent, concave-shaped devices directly placed on the ocular surface for correcting the refractive errors. Additionally, they are used as bandage for protecting the corneal epithelial layer, helping in healing, and sealing wound leaks (Al-Qahtani et al., 2014; Grinninger et al., 2015; Mohammadpour et al., 2015; Ubani-Ukoma et al., 2019). CLs are explored widely as drug carrier systems to enhance the residence time of the therapeutic molecules on the ocular surface. Various materials have been employed in fabricating CLs without effecting their oxygen permeability and light transmittance (Dubald et al., 2018; Garg et al., 2019; Wei et al., 2020). One of the major challenges in developing drug delivery systems is to optimize the relevant drug release kinetics to inhibit the microbes (Hui et al., 2014). Tan et al. (2016) reported the fabrication of drug loaded intra-ocular lens to enhance the bioavailability of the drugs. Levofloxacin solubilized in tetrahydrofuran during fabrication of intra-ocular lens showed sustained release of the drug compared to levofloxacin solubilized in dicholoromethane. The studies carried out using commercially available CLs displayed prolonged drug release (Karlgard et al., 2000). Hui et al. reported silicone hydrogel CLs using a molecular imprinting technique to optimize the release kinetics of ciprofloxacin. However, incorporation of ciprofloxacin during fabrication of CLs unfortunately decreased light transmission. Acrylic acid–modified CLs displayed significantly longer time for drug release than unmodified controls. *In vivo* studies showed that ciprofloxacin released by the modified CLs significantly inhibited *P. aeruginosa* infection than unmodified CLs (Hui et al., 2014). Kakisu et al. (2013) reported the fabrication of soft CLs for assessing their ability to uptake and release antibiotics for a better drug delivery system for treating keratitis. The prolonged release of drugs from the soft CLs was achieved by layer-by-layer (LbL) coating of polyelectrolytes (combinations of sodium alginate, chitosan, sodium hyaluronate, and poly-lysine hydrobromide) with drugs (Silva et al., 2018). LbL coating controlled the release of diclofenac sodium salt from CLs and enhanced antibacterial activity without any adverse effects. Molecular imprinting and LbL coating did not affect the properties of soft CLs, and drugs released from the lenses inhibited the growth of *S. aureus* and *S. epidermis* (Silva et al., 2021)*.* Ciolino et al. developed econzole impregnated poly-lactic-co-glycolic acid (PLGA) and poly-HEMA contact lenses for treating fungal keratitis. Econazole released from the contact lens efficiently inhibited the growth of *C. albicans* and displayed antifungal activity for up to three weeks. Huang et al. (2016) reported delivery of voriconazole using a hybrid CLs developed with quaternized chitosan, silver nanoparticles, and graphene oxide (HTCC/Ag/GO) for treating microbial keratitis. CLs are also used for coating with antimicrobial molecules and peptides and is one of the effective strategies for preventing or treating the microbial keratitis. Various studies have reported the coating of contact lenses with silver nanoparticles to treat microbial keratitis and showed inhibition of bacterial (Bazzaz et al., 2014; Shayani Rad et al., 2016) and fungal growth (Huang et al., 2016; Liu et al., 2018). However, the use of a high concentration of silver nanoparticles induced cytotoxicity to the mitochondrial activity, which became unsuitable for ophthalmic application (Bin Sahadan et al., 2019). Bin Sahadan et al. (2019) have reported the coating of contact lenses with phomopsidione nanoparticles, a ketone derivative, which excellently inhibited the growth of keratitis caused by Gram-negative bacteria. Melimine, a synthetic peptide derived from protamine and melittin, increased the hydrophilicity of the contact lenses and inhibited the growth of various microbes cultured on it (Dutta et al., 2013; Dutta et al., 2016a). It also inhibited *P. aeruginosa*–induced microbial keratitis in rabbit models (Dutta et al., 2016b). In a human trials, use of melimine-coated contact lenses increased mean corneal (depth, extent, and type) staining than control eyes (Dutta et al., 2014). Mel4, a peptide derived from melamine, also exhibited high antimicrobial activity toward *S. aureus* and *P. aeruginosa* as a contact lens coating material (Dutta et al., 2016a) and prevented the bacterial adhesion to contact lenses and displayed no sign of cytotoxicity toward rabbit models. During human trials, no sign of ocular difference was observed between test eyes (with Mel4-coated contact lenses) and the control eyes (Dutta et al., 2017).

### *In situ* Forming Hydrogels

Hydrogels are three-dimensional polymeric networks having high water-holding capacity, which could be made from natural or synthetic hydrophilic polymer (Torres-Luna et al., 2020). They are widely used for tissue engineering and drug delivery applications as they retain the bioactive molecules within the crosslinked matrices (Lynch et al., 2020). High viscosity of the hydrogels also enhances the residence time of the bioactive molecules at ocular surface by preventing their washout. Delivery of the bioactive molecules to a local site in a controlled manner make the hydrogels a suitable drug delivery system for ocular applications. Hydrogels are usually characterized as performed gels (achieved by crosslinking the polymers) or *in situ* gels (Sharma and Taniguchi, 2017; Kurniawansyah et al., 2018). Formation of gels at the site of interest by changing the pH or temperature or ion (electrolyte) composition after reacting with surround environment make the *in situ* gels advantageous for enhancing the bioavailability of drugs. Various studies have reported the use of *in situ* hydrogels to deliver antimicrobial agents for treating corneal infection. Shastri et al. (2010) have reported the formulation of thermoreversible mucoadhesive gels using poloxamer, xanthan gum, and sodium alginate to deliver moxifloxacin hydrochloride (MXF) for corneal application. *In vitro* studies showed that the *in situ* gels were transparent, had satisfactory adhesion to the sheep’s cornea, and sustained release of MXF (Shastri et al., 2010). In another study, MXF-loaded *in situ* hydrogels were formulated using sodium alginate and hydroxy propyl methyl cellulose (HPMC) for ocular application. *In vitro* studies showed the MXF released from the *in situ* gels inhibited bacterial growth of *E. coli* and *S. aureus*, whereas *in vivo* studies displayed that the gels were transparent and non-irritating with no ocular damage (Mandal et al., 2012). El-Laithy et al. (2011) reported the preparation of *in situ* gels based on Gelrite to deliver MXF for treating bacterial keratitis. MXF-loaded gels delivered higher amount of drug into ocular region in comparison to Vigamox^®^ commercial eye drops without causing ocular irritation and efficiently reduced bacterial keratitis (El-Laithy et al., 2011). Nair et al. (2021) also reported the development of *in situ gel* formulation by optimizing the concentration of polymers like gellan gum, sodium alginate, and HPMC for enhancing bioavailability and efficiency of MXF. Selected formulation significantly improved the bioavailability of MXF to the ocular region when compared to commercially available eye (Nair et al., 2021). Mohammed et al. (2017a) reported formulation of MXF or gentamicin-loaded chitosan/β-glycerolphosphate *in situ* gels for treating bacterial keratitis. Gels displayed sustained cumulative release of antibiotics that inhibited the growth of *S. aureus* and also protected the corneal fibroblast from bacteria-induced death (Mohammed et al., 2017b). Gatifloxacin was also delivered using ion-activated mucoadhesive *in situ* hydrogels (combination of sodium alginate, or gellan, and sodium carboxymethylcellulose) to treat bacteria keratitis in rabbit models (Kesavan et al., 2016). *In vivo* studies showed that ion-activated mucoadhesive hydrogels were non-irritants without causing inflammatory reactions and gatifloxacin released from the gels significantly reduced bacterial infection in comparison with marketed drug solution.

### Microneedle Ocular Patch

Delivery of effective therapeutic to internal ocular regions are limited due to ocular barriers like cornea epithelium. Drugs delivered using contact lenses enhance the retention time in the ocular surface; however, penetration of drug into ocular regions is questionable. These problems are addressed using microneedle ocular patch. Microneedles ocular patch haves an array of 50–1,000 µm sized needles, which are fabricated using ceramic, metals, polymer, and silicon (Garg et al., 2019). The microneedle ocular patches containing amphotericin have also been found to be effective in treating fungal keratitis in rabbits (Roy et al., 2019). Microneedle could deliver drugs into the corneal stroma by bypassing tear films and penetrating the corneal epithelium (Lee et al., 2018). Several studies have reported the delivery of bioactive molecules or drugs using microneedle ocular patch for various applications, including keratitis. Bhatnagar et al. reported the fabrication of microneedles using polyvinyl pyrrolidone (PVP) and polyvinyl alcohol (PVA) by micromolding technique to deliver besifloxacin for treating microbial keratitis. Besifloxacin delivered into cornea by dissolution of microneedles showed enhanced antibacterial activity and reduced *S. aureus* growth in the *ex vivo* corneal infection model (Bhatnagar et al., 2018). In another study, Lee et al. developed a detachable hybrid microneedle pen for delivering therapeutics to treat keratitis infections. Polyhexamethylene biguanide released into the cornea from detached poly(lactic-coglycolic) acid (PLGA) microneedle showed effective inhibition of *Acanthamoeba*-induced keratitis in mice models (Lee et al., 2018).

### Nanocarriers-Based Drug Delivery

Nanotechnology bridges a barrier between the biology and physical sciences by developing nanostructure using synthetic or natural polymers to address various problems in the field of sciences (Patra et al., 2018). Nanocarriers synthesized from biocompatible and biodegradable natural or synthetic polymers overcome corneal barriers and deliver the drug to deeper ocular regions that reduce drug toxicity and precorneal drug loss (Gote et al., 2019). Various studies have been reported regarding delivery of drugs using different nanocarriers for treating ocular disorders including keratitis. Micelles are good drug carriers that entrap the drug in the hydrophobic core; whereas, hydrophilic shells form a steric barrier and prevents micelles aggregation (Guo et al., 2020). Jaiswal et al. reported the development of micelles to deliver itraconazole for treating fungal keratitis. Itraconazole released from itraconazole-loaded micellar *in situ* gel showed greater antifungal activity than commercial eye drops (Jaiswal et al., 2015). Sayed et al. (2018) enhanced the penetration of itraconazole into the deeper cornea of eyes using β-cyclodextrin consolidated micellar dispersions (CCMD) to treat fungal keratitis *ex vivo* and *in vivo* models. In another study, micelles were synthesized using self-assembled poly(ethylene glycol)-block-poly(glycidyl methacrylate) (PEG-b-PGMA) to deliver natamycin for treating fungal keratitis (Guo et al., 2020). Drug-loaded micelles were nontoxic, and the natamycin released from the micelles suppressed the growth of *C. albicans* in rabbit models. Nanoparticles are colloidal carrier systems made of natural or synthetic polymers and stores the drug in the polymer shell and deliver them to the target site (Garg et al., 2019). Ahsan et al. reported the development of anti-TLR4 antibodies–conjugated gelatin nanoparticles for enhancing the residence time of the drug ketoconazole by anchoring to the cornea. Released drug exhibited the reduction of corneal inflammation and inhibited the growth of *A. flavus* in rat infection models (Ahsan and Rao, 2017). Lecithin/chitosan mucoadhesive nanoparticles used or enhancing the drug retention time in ocular regions enhanced the bioavailability and resident time of amphotericin-B in the corneal region of rabbit eyes. *In vitro* studies displayed that amphotericin-B released from nanoparticles inhibited the growth of *C. albicans* and *A. fumigatus* more effectively than marketed formulations (Chhonker et al., 2015). Shivam et al. (2020) reported the synthesis of MXF-loaded nanoparticles entrapped in *in situ* gel to demonstrate sustained drug release to improve efficacy of drug in treating bacterial keratitis; reduced bacterial load and recovery of corneal epithelium was observed in *in vivo* studies treated with MXF-loaded nanoparticles. Nanostructured lipid nanocarriers (NLCs) are new generation formulations of solid lipid nanoparticles, which are used as an alternative for conventional drug delivery. Üstündağ-Okur et al. (2014) reported development of ofloxacin-loaded NLCs to treat bacterial keratitis. Modification of ofloxacin-loaded NLCs with chitosan enhanced the preocular residence time of the nanocarrier. Ofloxacin delivery by ofloxacin-loaded NLCs-chitosan showed a significantly higher rate of penetration into cornea than commercial solution. Niosome are non-ionic surfactant bilayer vesicles that are formed by the self-assembly of non-ionic surfactants in aqueous environment, also acts as a potential carrier to deliver drug for treating ocular disorders (El-Nabarawi et al., 2019). They also showed natamycin-loaded niosomes entrapped in ketorolac tromethamine gels enhance the clinical efficacy of drug by improving its penetration of corneal tissue and minimizing inflammation associated with microbial keratitis. Natamycin delivered from the formulation inhibited the *Candida albicans* growth in the rabbit model and KT gels minimized the corneal inflammation caused by fungus (El-Nabarawi et al., 2019). In another study, El-Mofty et al. (2020) reported the development of new formulation by modifying natamycin-loaded nanoparticle niosomal formulation along with KT to improve the safety and efficacy in fungal keratitis. Newly developed F1 (natamycin-loaded nanoparticle niosomes/0.5% KT, 4% carboxymethyl cellulose gel) formulation showed higher viscosity and mucoadhesive property than F2 formulation (natamycin-loaded nanoparticle niosomes/0.5% KT, 2% hydroxypropylmethyl cellulose-E4 gel). F1 formulation also enhances the penetration of drug-loaded niosomal into cornea and increased the bioavailability of the natamycin that resulted in inhibition inflammation and growth of *Aspergillus* spp. .

## Conclusion

Currently, there is an alarming increase in antibiotic resistance and is considered as a global concern that requires more focused research to develop alternative therapeutic interventions to battle the growing trend. The AMR is increasing among ocular pathogens as well and impacting patient care to a large extent. The studies present in this review highlight the significant role of alternative therapeutics to combat increasing antimicrobial resistance and AMPs are explored widely and considered as the most potent new age therapeutics. The challenge has given us an opportunity to explore new antimicrobial molecules, repurpose existing molecules, explore alternative nonantibiotic therapies, and the drug delivery system that ensure concentration effective against drug-resistant pathogens. AMPs are one of the most potent alternatives that can be considered to fight antibiotic resistance as in addition to direct microbial killing activity, and it also plays an integral role in innate immune system. The small molecules targeting the virulence factors of the pathogens are also emerging as promising alternatives as they are not inhibiting microbial growth and generating selective pressure. These alternative interventions provide hope and feasible option for developing AMPs, small molecules, and natural compound analogues as future generation antimicrobials to combat antibiotic resistance.
